# Retracted: Mechanism of Hepatoprotective Effect of *Boesenbergia rotunda* in Thioacetamide-Induced Liver Damage in Rats

**DOI:** 10.1155/2018/1072403

**Published:** 2018-07-18

**Authors:** Evidence-Based Complementary and Alternative Medicine

Evidence-Based Complementary and Alternative Medicine has retracted the article titled “Mechanism of Hepatoprotective Effect of *Boesenbergia rotunda* in Thioacetamide-Induced Liver Damage in Rats” [1]. Figure 5(a)-(iv) is reused from Figure 15A-II in an article by the same authors, Salama et al. [2].

An institutional investigation by the University of Malaya found there was no system to index and file data and images to avoid mislabeling and mishandling, which led to errors and duplication of research data. The authors did not thoroughly check the manuscript before submission.

The authors said that to save time, animal experiments were conducted on both plants at the same time and to cut costs, immunohistochemistry staining was performed at the same time on the liver tissues collected from all animals of both experiments using one kit and one protocol. In addition, the results of both experiments were very close and so they mixed up images between the two experiments.
